# Tissue-specific amino acid accumulation in *Cynomorium songaricum* stem and epidermis responds to distinct water and soil cues

**DOI:** 10.1371/journal.pone.0354162

**Published:** 2026-07-24

**Authors:** Yu Tang, Xunchao Zhang, Yang Yang, Wenshu Wang, Jie Wang, Yubi Zhou

**Affiliations:** 1 Qinghai Provincial Key Laboratory of Tibetan Medicine Pharmacology and Safety Evaluation, Northwest Institute of Plateau Biology, Chinese Academy of Sciences, Xining, China; 2 School of Pharmacy, Qinghai Minzu University, Xining, China; 3 University of Chinese Academy of Sciences, Beijing, China; 4 Qinghai Provincial General Station of Forest Seed and Seedling, Xining, China; 5 Alxa League Institute of Forestry and Grassland, Bayanhot, China; Universidade do Minho, PORTUGAL

## Abstract

This study provides the first evidence, from the perspective of intra-organ metabolic division of labour, that *Cynomorium songaricum* adapts to the heterogeneity of desert habitats through a synergistic strategy of “stem epidermis perceiving the water environment and fleshy stem buffering soil stress.” The research found a significant spatial metabolic division within the organs of *C. songaricum*: the stem epidermis is enriched in various essential and functional amino acids, including threonine and serine, with their content significantly higher than in the fleshy stem. In contrast, the fleshy stem specifically accumulates proline and cysteine, showing a significant difference. Environmental driving analysis indicates that, although *C. songaricum* is a holoparasitic root parasite, the metabolic division within its organs is still significantly driven by differential rhizosphere soil-water environmental factors: the amino acid profile of the stem epidermis is sensitive to water environmental factors such as electrical conductivity and carbonate ions, showing a positive correlation; whereas the amino acid composition of the fleshy stem is mainly regulated by soil factors such as water-soluble salts, showing a negative correlation. This suggests that, even while relying on the host for nutrition, the metabolic characteristics of *C. songaricum* are still influenced by its growth micro-environment. This finding breaks through the research paradigm of treating plants as homogeneous entities, providing new insights into understanding plant ecological adaptability and offering a key scientific basis for the artificial adaptive management and comprehensive utilization of *C. songaricum* resources.

## 1. Introduction

*Cynomorium songaricum* Rupr., a perennial succulent parasitic herbaceous plant belonging to the *Cynomorium* genus of the Cynomoriaceae family, is a characteristic species of the arid desert ecosystem in northwestern China [1]. It is one of the few species capable of thriving in environments with extreme aridity, high salinity, and diurnal temperature variations exceeding 30°C, primarily distributed in provinces and regions such as Inner Mongolia, Xinjiang, and Qinghai [2]. This unique survival strategy has made it an ideal model for studying biological adaptation [3]. However, due to factors such as habitat vulnerability, over-harvesting, and difficulties in natural propagation, the survival of this species is facing serious threats [4,5]. *C. songaricum* is not only listed as a National Second-Class Key Protected Wild Plant in China but is also included as Vulnerable on the IUCN Red List of Threatened Species and receives Appendix II protection under CITES [6].

*C. songaricum* is a plant with medicinal and edible properties [7]. Its medicinal value is associated with the diverse active compounds contained in its fleshy stem, among which amino acids are particularly important [8]. In plant physiological ecology, amino acids serve not only as building blocks for protein synthesis but also as key participants in stress physiology [9,10]. They function as osmoregulators to maintain cellular water balance, act as precursors for defensive secondary metabolites, and serve as antioxidants to mitigate oxidative damage caused by environmental stressors such as drought and high ultraviolet radiation [11–13]. Therefore, the regulation of amino acid metabolism is a dynamic adaptation process directly influenced by environmental stress. Although *C. songaricum* is a holoparasitic root parasite that relies on its host for nutrient acquisition, this study reveals that its amino acid metabolic profile is still significantly and directly regulated by rhizosphere soil and water environmental factors. This indicates that the plant is not entirely detached from environmental influences; rather, its physiological state remains highly responsive to micro-environmental changes [14,15].

Currently, most studies treat *C. songaricum* as a homogeneous entity for analysis, an approach that fails to capture its response mechanisms in real-world environments [16,17]. From a plant physiological perspective, the fleshy stem, which serves a storage function, and the stem epidermis, which acts as the primary interface for environmental interaction, are likely to possess distinct metabolic profiles [18]. As the first line of defense against environmental stress, the composition of secondary metabolites in the stem epidermis may exhibit unique environmental response patterns [19]. Ignoring tissue-level metabolic differentiation precludes systematic elucidation of the mechanism by which *C. songaricum* adapts to extreme environments. While carbohydrates modulate plant water status [20], amino acids play irreplaceable roles in resisting multiple abiotic stresses. They mediate osmotic adjustment, reactive oxygen scavenging, signal transduction, and secondary metabolism synthesis. As an obligate parasite unable to select habitats freely, *C. songaricum* adapts to harsh desert environments largely via flexible amino acid metabolism [21]. Accordingly, this study focuses on amino acid profiles to reveal tissue metabolic differentiation and environmental response rules.

Based on this, this study proposes the hypothesis that significant differences exist in the amino acid composition between the fleshy stem and stem epidermis of *C. songaricum*, and that the stem epidermis, as an interface organ, responds to soil and water environmental factors more sensitively and uniquely than the fleshy stem. To test this hypothesis, this study systematically collected tissue samples from 33 geographical populations of *C. songaricum*, along with corresponding water and soil samples. Our objectives are: (1) to clarify the distribution patterns of amino acids across different tissues; (2) to elucidate the spatial variation patterns of rhizosphere environmental factors; and (3) to identify the key environmental drivers responsible for the differences in amino acids between different tissues. By integrating environmental factors with tissue-specific metabolic profiles, this study provides a new perspective for understanding the ecological adaptation of this rare species and offers a scientific basis for its precise artificial cultivation and resource conservation.

## 2. Materials and methods

### 2.1. Study site and sample collection

The experimental materials consisted of fresh *C. songaricum* Rupr. Plants at the “outcrop stage” were collected from 33 sampling sites in the Alxa region of Inner Mongolia Autonomous Region between July and August 2019. The study was conducted in the Alxa region, Inner Mongolia, China. A total of 33 sampling sites were selected to cover the main distribution area of *C. songaricum*. No specific permits were required for the collection of *C. songaricum* samples in the Alxa region, because at the time of collection, this species was not protected under local wildlife protection regulations in that area. All collection work complied with the relevant laws and regulations of China.

At each site, three healthy individuals with consistent growth phenotypes and no visible disease spots or insect damage were selected. Species identification was performed by Professor Zhou Yubi from the Northwest Institute of Plateau Biology, Chinese Academy of Sciences. On the day of sampling, three types of samples were collected simultaneously: the fleshy stems of *C. songaricum*, rhizosphere soil, and surrounding water samples. The specific procedures were as follows:

The fleshy stems of *C. songaricum* were cleaned of surface impurities, such as sediment, and then partitioned. Rhizosphere soil samples were collected using a sterile soil auger at a depth of 20–40 cm after removing non-soil impurities such as fallen leaves and stones. A 500 mL groundwater sample was collected from local herder wells near each sampling site, sealed, and preserved.

The collected *C. songaricum* stems were peeled with a sterile knife to separate the epidermis from the internal fleshy tissue. The epidermis and internal fleshy stems were then packaged separately, transported to the laboratory on the same day, and immediately frozen at −80°C for subsequent determination of amino acid composition and content. The soil and water samples were transported to the laboratory on the same day and stored at −4°C for physicochemical property analysis.

All samples were assigned unique identification numbers (S1–S33) corresponding to the 33 sampling sites. Detailed geographic coordinates, elevation, and host plant information are provided in Supplementary S1 Table.

### 2.2. Soil and water analysis

#### 2.2.1. Sample analysis parameters.

The analysis of the collected rhizosphere soil and water samples was conducted by the National Forest and Grassland Administration Economic Forest Product Quality Inspection and Testing Center (Hangzhou). The following parameters were determined for the rhizosphere soil samples: As (mg/kg), pH, electrical conductivity (μS/cm), Ca (%), Cd (mg/kg), Cr (mg/kg), Al (%), Mg (%), Na (%), Pb (mg/kg), total nitrogen (%), total potassium (%), total phosphorus (%), water-soluble calcium (mg/kg), water-soluble potassium (mg/kg), water-soluble sodium (mg/kg), water-soluble salts (mg/kg), available potassium (mg/kg), Fe (%), organic matter, available phosphorus (mg/kg), Co (mg/kg), Ti (%), Hg (mg/kg), ammonium nitrogen (mg/kg), chloride ion (mg/kg), and nitrate nitrogen (mg/kg).

For the water samples, the following parameters were determined: pH, electrical conductivity (μS/cm), Ca (mg/L), K (mg/L), Na (mg/L), Mg (mg/L), Cr (mg/L), As (mg/L), total dissolved solids (mg/L), carbonate ion (mg/L), bicarbonate ion (mg/L), fluoride ion (mg/L), phosphate ion (mg/L), sulfate ion (mg/L), chloride ion (mg/L), and nitrate ion (mg/L). Soil and water physicochemical properties were determined in accordance with Chinese national standards, industrial standards, and authoritative monographs. Detailed detection methods are provided in Supplementary S2–S3 Tables.

#### 2.2.2. Equipment and chemicals.

The analysis employed the following instruments: a Metrohm Ion Chromatograph (YLSZJ-SB-293, Switzerland), an Inductively Coupled Plasma Mass Spectrometer (YLSZJ-SB-195, China), a pH Meter (YLSZJ-SB-014, Switzerland), a Conductivity Meter (YLSZJ-SB-215, Switzerland), a Nitrogen Determination Apparatus (YLSZJ-SB-007), an Inductively Coupled Plasma Optical Emission Spectrometer (YLSZJ-SB-235, USA), a Gas Chromatograph (YLSZJ-SB-227, Japan), a QDN-Ⅱ Fully Automatic Kjeldahl Nitrogen Analyzer from Hangzhou Huier Instrument Equipment Co., Ltd. (China), a Biochrom 30+ Amino Acid Analyzer from DKSH Commerce (China) Co., Ltd. (China), a Molement Element Ultra-Pure Water System from Shanghai Mole Biotechnology Co., Ltd. (China), and an Electric Thermostatic Drying Oven from Guangzhou Kangheng Instrument Co., Ltd. (China).

The reagents used included Hydrochloric Acid (Guaranteed Reagent) and Sulfuric Acid (Analytical Grade) from Chengdu Kelong Chemical Co., Ltd.; Anhydrous Ethanol (Analytical Grade) from Tianjin Komiou Chemical Reagent Co., Ltd.; Phenol, Sodium Citrate pH Buffer, and Ninhydrin (Guaranteed Reagent) from Tianjin Xinbote Chemical Co., Ltd.; and a 17 Amino Acid Mixed Standard Solution (2.5 µmol/mL) from DKSH Commerce (China) Co., Ltd.

### 2.3. Amino acid quantification of *C. songaricum* samples

Following portioning, 100 g of the stem epidermis and internal fleshy stem of *C. songaricum* were randomly selected from each sample and mechanically homogenized for further use. A 0.5 g portion of the powdered sample was weighed into a hydrolysis tube, followed by the addition of 10 mL of 6 mol/L hydrochloric acid solution and 2 drops of phenol. The tube was sealed under a nitrogen atmosphere and placed in an electric thermostatic blast drying oven at 103 °C for 22 hours. After hydrolysis, the tube was removed and cooled to room temperature. The hydrolysate was filtered into a 50 mL volumetric flask. The hydrolysis tube was rinsed with pure water, and the rinsate was combined into the same volumetric flask. The solution was then brought to volume and mixed thoroughly by shaking. Exactly 1 mL of the filtrate was pipetted into a test tube and dried under reduced pressure at 40 °C. The residue was dissolved in 1 mL of pure water and subjected to further drying under reduced pressure. Finally, the residue in the tube was dissolved in 1 mL of sodium citrate buffer solution (pH = 2.2), vortex-mixed, and filtered through a 0.22 μm membrane to obtain the test solution for instrumental analysis.

A sulfonic acid-based cation-exchange chromatography column was used for the analysis. The detection wavelengths were set at 570 nm and 440 nm. A sodium citrate-citric acid buffer was used as the mobile phase with a flow rate of 0.25 mL/min. The separation column (15 cm in length) was maintained at 53 °C, with a column pressure ranging from 9.7 to 10.1 Pa. The ninhydrin solution was delivered at a flow rate of 0.3 mL/min, with a pump pressure maintained between 80 and 90 kgf/cm^2^. The reaction coil temperature was set at 98 °C. An injection volume of 20 μL was used, and the total analysis time was 45 minutes.

### 2.4. Statistical analysis

The data analysis was performed using the following software tools: Microsoft Excel 2021 was used for organizing and performing basic statistical calculations on the soil property and amino acid content data. Origin 2021 was employed to generate the box plots illustrating the amino acid contents of *C. songaricum* and the principal component analysis (PCA) score plots for the soil physicochemical properties. SPSS Statistics 26.0 was utilized to calculate the contribution rates of the principal components for the soil physicochemical factors. Based on the R language environment, Z-score normalized heatmaps for the water and soil environmental factors, as well as a correlation network analysis diagram between environmental factors and amino acids, were constructed.

The climatic factors used in this study were obtained from the National Tibetan Plateau Data Center (https://data.tpdc.ac.cn/home). The map boundaries of sampling sites were derived from the National Geomatics Center of China – Tianditu (https://www.tianditu.gov.cn/). The elevation data were acquired from the Geospatial Data Cloud (https://www.gscloud.cn/search). All environmental and spatial data were preprocessed uniformly before being used in correlation network analysis and spatial heterogeneity tests of samples, so as to reveal the spatial correlation characteristics among different geographical populations.

## 3. Results

### 3.1. Distribution of amino acids in different tissues of *C. songaricum*

**Fig 1:**
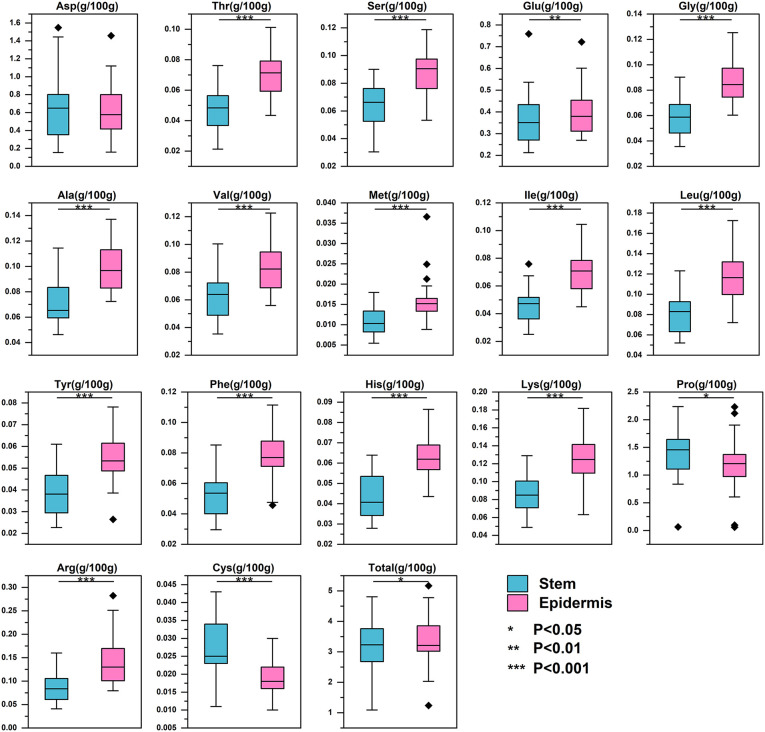
Box plots of 17 free amino acids and total amino acid contents in the fleshy stem and the stem epidermis of *Cynomorium songaricum.* Note: Stem denotes fleshy stem, and Epidermis denotes stem epidermis; *, **, and *** indicate significant differences at *P* < 0.05, *P* < 0.01 and *P* < 0.001, respectively; (a) shows the distribution of the 33 sampling sites, and (b, c) show representative *C. songaricum* specimens from the Alxa region.

To investigate the tissue-specific differences in amino acid content between the stem and stem epidermis of *C. songaricum* (Supplementary S4–S5 Tables), we performed a box plot analysis of 17 free amino acids and total amino acids in both tissues. Overall, all measured amino acids except proline (Pro) and cysteine (Cys) (which were significantly higher in the stem) and aspartic acid (Asp) (which showed no significant difference) were significantly or highly significantly more abundant in the stem epidermis than in the fleshy stem (Fig 1). The detailed comparisons are as follows.

The total amino acid content in the stem epidermis was significantly higher than that in the stem. Specifically, the contents of threonine (Thr), serine (Ser), glycine (Gly), alanine (Ala), valine (Val), methionine (Met), isoleucine (Ile), leucine (Leu), tyrosine (Tyr), phenylalanine (Phe), histidine (His), lysine (Lys), and arginine (Arg) were all significantly higher in the epidermis. The content of glutamic acid (Glu) was also significantly higher in the epidermis. In contrast, the stem exhibited a significantly higher content of proline (Pro) than the epidermis, and a significantly higher content of cysteine (Cys). Only aspartic acid (Asp) showed no significant difference between the stem and the stem epidermis.

In summary, the stem and stem epidermis of *C. songaricum* exhibited distinct tissue specificity in their amino acid composition and content. The stem epidermis serves as an enrichment site for most amino acids, particularly essential and functional amino acids, while the stem possesses a higher abundance of proline and cysteine. This tissue-specific distribution pattern may be related to their physiological functional differentiation and adaptation strategies to the desert environment.

### 3.2. Principal component analysis of soil and water environmental factors

**Fig 2:**
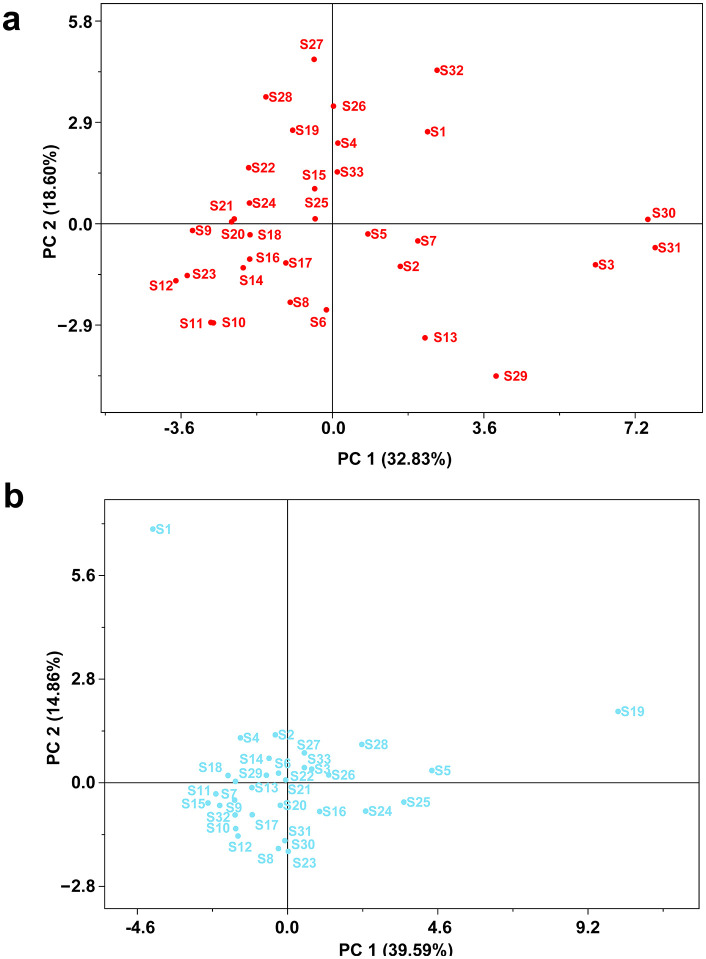
Principal component analysis (PCA) of soil and water environmental factors. Note: (a) PCA score plot of soil environmental factors; (b) PCA score plot of water environmental factors. S1–S33 represent rhizosphere samples of *Cynomorium songaricum* from 33 different geographic populations.

Principal component analysis (PCA) was performed on soil and water environmental factors to investigate their spatial variability (Fig 2; Supplementary S6–S7 Tables). For the soil factors (Fig 2a), four principal components (PC1–PC4) were extracted, collectively explaining 70.55% of the total variance. To identify the main driving factor for each principal component, we selected the environmental variable with the highest absolute loading value within that component (i.e., the maximum loading criterion). Among these, PC1 (32.83% of variance) was primarily driven by water-soluble salts; PC2 (18.60%) was dominated by Soil_Fe; while Soil_Al and Soil_Co were the highly loaded variables for PC3 (11.64%) and PC4 (7.47%), respectively (Table 1). The principal component score plot illustrated the distribution pattern of the 33 geographic populations in the PC1–PC2 space, reflecting the spatial heterogeneity in soil factor combinations. Populations S1 and S19 were clearly separated from others in the score plot, suggesting that the combined characteristics of soil water-soluble salts and iron content in these regions differ significantly from those in other areas.

**Table 1:** Variance contribution rates of principal components for soil and water environmental factors.

Medium Type	Principal Component	High-Loading Variables	Eigenvalue	Variance Contribution Rate	Cumulative Contribution Rate
Soil	PC1	Water-soluble salts	8.86426	32.83%	32.83%
	PC2	Fe	5.02282	18.60%	51.43%
	PC3	Al	3.14323	11.64%	63.08%
	PC4	Co	2.01728	7.47%	70.55%
Water	PC1	EC	6.3345	39.59%	39.59%
	PC2	As	2.37714	14.86%	54.45%
	PC3	CO_3_^2^	1.92291	12.02%	66.47%
	PC4	pH	1.53453	9.59%	76.06%

For the water environmental factors, four principal components (PC1–PC4) were also extracted, with a cumulative variance contribution rate of 76.06%. Using the same maximum loading criterion, EC (electrical conductivity) was the key driving factor for PC1 (39.59% of variance), As (arsenic content) drove PC2 (14.86%), CO_3_^2−^ (carbonate ion) drove PC3 (12.02%), and pH drove PC4 (9.59%) (Table 1). The principal component score plot (Fig 2b) displayed the distribution of the 33 geographic populations in the PC1–PC2 space, indicating spatial differentiation in water environmental factor combinations. For instance, populations S27, S28, and S32 clustered together in the score plot, while S29 was distinctly separated, reflecting grouping differences in hydrochemical properties—such as ionic strength and arsenic content—among different regions.

In summary, these principal components captured most of the variability in the soil and water environments, laying a foundation for subsequent analysis of the driving mechanisms of environmental factors on the amino acid distribution in *C. songaricum*.

### 3.3. Correlation analysis between environmental factors and amino acids

**Fig 3:**
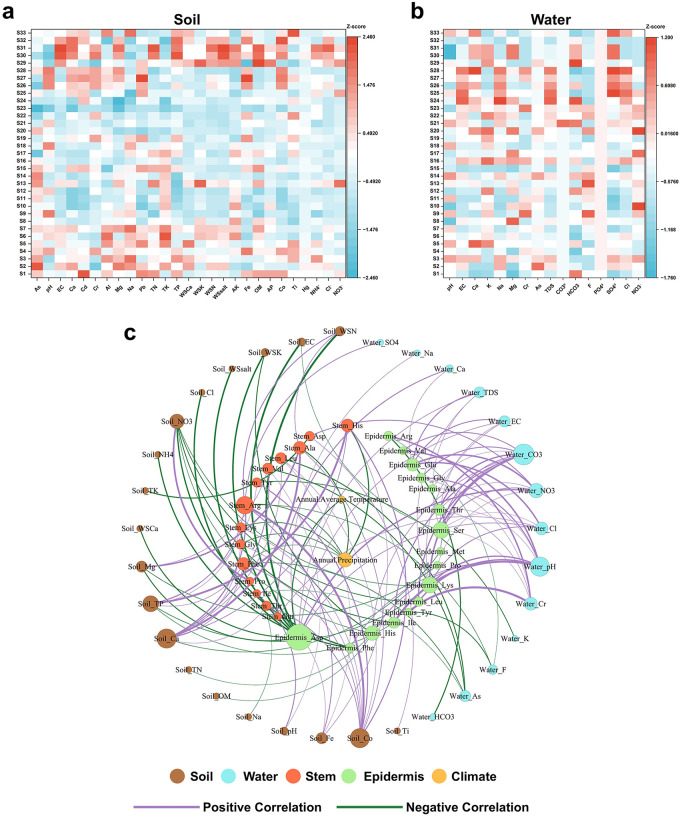
Environmental factor heatmaps and correlation network analysis of *Cynomorium songaricum.* Note: Correlations of *C. songaricum* amino acids with environmental factors. (a) Z-score heatmap of soil environmental factors across the 33 geographic populations; (b) Z-score heatmap of water environmental factors across the 33 geographic populations; (c) Correlation network showing significant associations (|r| ≥ 0.5, P < 0.05). Purple edges indicate positive correlations, green edges indicate negative correlations. Abbreviations: Soil indicators: As arsenic, Cd cadmium, Cr chromium, Al aluminum, Mg magnesium, Na sodium, total potassium, total nitrogen, total phosphorus, water-soluble calcium, water-soluble potassium, water-soluble sodium, water-soluble salts, available potassium, Fe iron, organic matter, available phosphorus, Co cobalt, Ti titanium, Hg mercury, ammonium nitrogen, chloride ion, nitrate nitrogen; Water indicators: electrical conductivity, Ca calcium, K potassium, Na sodium, Mg magnesium, Cr chromium, As arsenic, total dissolved solids, carbonate ion, bicarbonate ion, fluoride ion, phosphate ion, sulfate ion, chloride ion, nitrate ion; Climate factors: T average temperature, Pre annual precipitation.

Using heatmaps and correlation network analysis, this study systematically investigated the associations among soil, water, environmental, and climate factors and the amino acid contents in the stem and rind of *C. songaricum*. The Z-score heatmaps of soil factors (Fig 3a) and water environmental factors (Fig 3b) visually displayed the spatial variation patterns of each environmental factor across the 33 geographic populations. Both soil and water environmental factors exhibited significant spatial heterogeneity across populations, providing the context for environmental variation in the subsequent correlation analysis.

Pearson correlation coefficients were calculated between all environmental factors (soil, water, and climate) and amino acids in the stem epidermis and fleshy stem. For network visualization (Fig 3c), only correlation pairs with |r| ≥ 0.5 and P < 0.05 were retained. The correlation network analysis (Fig 3c) further revealed differential association patterns between environmental factors and tissue-specific amino acids. Soil factors showed significant negative correlations with most amino acids in the fleshy stem, indicating that high salinity and high ionic strength inhibit amino acid accumulation in the fleshy stem. This may represent an adaptive strategy of *C. songaricum* to cope with salt stress, reduce intracellular osmotic pressure, and alleviate ion toxicity. In contrast, water factors exhibited significant positive correlations with various amino acids in the stem epidermis, suggesting that the epidermis, as the outermost interface tissue, is highly sensitive to hydrochemical characteristics and can achieve environmental perception and defense responses by enriching functional amino acids. Meanwhile, climatic factors were also significantly correlated with multiple amino acids in both tissues, implying that large-scale climatic conditions jointly regulate amino acid metabolism together with soil and water factors.

In summary, these distinct correlation patterns clearly reveal the tissue-specific environmental response strategy of *C. songaricum*: amino acids in the fleshy stem are mainly regulated by soil salinity to maintain osmotic balance and redox homeostasis, whereas amino acids in the epidermis are primarily driven by water conditions to support environmental perception and stress defense. The physiological functional differentiation between the two tissues further elucidates the core mechanism by which *C. songaricum* adapts to heterogeneous desert habitats through amino acid metabolic division of labor.

## 4. Discussion

This study, by integrating analyses of tissue-specific amino acid metabolism and environmental factors, systematically reveals the intrinsic strategy employed by *C. songaricum* to adapt to the extreme desert environment: the formation of a distinct metabolic division of labor at the organ level and a differential responsiveness to rhizosphere environmental signals. This finding challenges the conventional research paradigm that treats *C. songaricum* as a homogeneous entity and provides a fresh perspective for understanding the ecological adaptation mechanisms of rare parasitic plants.

### 4.1. Amino acid metabolism and ecological adaptation

This study reveals that *C. songaricum* has developed a sophisticated internal strategy at the organ level to cope with the extreme desert environment. Data indicate that the contents of 13 amino acids—including Thr, Ser, and Phe—in the stem epidermis are significantly higher than those in the fleshy stem, with an average increase of 42.7%. This suggests that the stem epidermis is far from being a simple protective tissue but rather serves as an active “frontier for defense and signal perception.” The enrichment of functional amino acids such as threonine and serine may provide the molecular basis for constructing complex defense metabolic networks, including glycosylation modifications and stress signal transduction [22]. Meanwhile, aromatic amino acids like Tyr and Phe, which act as precursors for UV-screening compounds and alkaloids, directly enhance the physical barrier function against intense radiation and biotic stress [23,24]. This epidermal specialization represents a frontline sensing and defensive module that enables rapid detection and response to fluctuating desert microenvironments.

Notably, Asp showed no tissue difference but was strongly regulated by environmental factors in the epidermis. As a central precursor for multiple functional amino acids, Asp maintains stable content while its metabolism is highly sensitive to soil–water cues [25,26]. In contrast, the fleshy stem has evolved into an efficient internal homeostasis maintenance system. The proline content in the fleshy stem is 28.3% higher than that in the stem epidermis, which not only contributes to classical osmotic regulation to maintain cell turgor pressure [11], but also plays a critical role in stabilizing protein structures as a molecular chaperone. This function is essential for the water-storage organ to cope with drastic fluctuations in internal and external water conditions [27]. More importantly, the significant accumulation of cysteine in the fleshy stem highlights its role as a strategic hub in the antioxidant defense system of *C. songaricum* [28]. As a precursor for glutathione synthesis, high cysteine levels ensure a robust redox buffering capacity, enabling the plant to mitigate oxidative damage induced by intense light and drought [29].

In summary, the highly specific differentiation in amino acid metabolism between the stem epidermis and fleshy stem of *C. songaricum* is not coincidental but represents an evolutionary adaptation strategy to multiple stressors in desert habitats. This strategy involves internal division of labor and synergistic adaptation: the stem epidermis specializes in environmental perception and immediate defense, while the fleshy stem focuses on osmotic regulation and long-term maintenance of redox homeostasis. Such functional complementarity and optimized resource allocation between organs significantly enhance the overall adaptability and survival probability of *C. songaricum* in harsh environments.

### 4.2. Environmental factors and their linkages to amino acids

This study further elucidates the significant functional divergence between the stem epidermis and fleshy stem of *C. songaricum* in response to rhizosphere environmental factors [30]. Principal component analysis indicated that soil and water environmental factors collectively form the key environmental context influencing the metabolism of *C. songaricum*, with soil water-soluble salts and iron content, as well as water electrical conductivity and carbonate ions, being the dominant factors driving the variation in soil and water environments, respectively.

Correlation network analysis revealed that amino acids in the stem epidermis showed significant positive correlations with water electrical conductivity and carbonate ions, suggesting that the stem epidermis, as an environmental interface tissue, may rapidly respond to hydrochemical changes through osmotic or epidermal signaling pathways [31,32]. In contrast, amino acids in the fleshy stem were mainly negatively regulated by soil water-soluble salts, reflecting their physiological role as a storage organ in mitigating soil osmotic stress through the accumulation of proline and cysteine [33]. This differential response mechanism reflects a synergistic adaptation strategy developed by *C. songaricum* at the organ level: “stem epidermis perceives water environment, while fleshy stem buffers soil stress.” This dual modular response allows the holoparasite to maintain metabolic autonomy despite relying on hosts for nutrients, thereby finely tuning its physiology to heterogeneous soil–water conditions. Future studies could integrate transcriptomic and enzymatic activity analyses to further unravel the underlying molecular regulatory network and construct a trinity ecological adaptation model of “perception-storage-regulation,” providing a theoretical framework for understanding resource allocation and metabolic division of labor in plants within heterogeneous habitats [34–36].

### 4.3. Research significance and conservation implications

The innovation of this study lies in the analysis of plant ecological adaptability at the internal substructural level of plant organs. It preliminarily explores that the “spatial metabolic division of labor of amino acids” may be an important mechanism for *C. songaricum* to respond to environmental heterogeneity [37,38]. Based on large-sample data from 33 geographic populations and multivariate statistical analysis, we have demonstrated the significant value of intra-organ specificity studies in elucidating plant environmental adaptability. This understanding not only holds theoretical importance for plant stress ecology but also provides clear guidance for the conservation of *C. songaricum* resources [3]. It is worth noting that this study mainly focuses on amino acid metabolism without systematically considering other metabolites closely related to plant water status, such as carbohydrates and organic acids. Therefore, future research can further integrate various metabolites, including sugars and inorganic ions, to construct a tissue-specific global metabolic regulatory network, so as to more comprehensively and systematically clarify the adaptation strategies of *C. songaricum* to extreme environments. In terms of resource conservation practice, fine regulation of the rhizosphere microenvironment can be carried out and differentiated management can be implemented according to the metabolic characteristics of different plant tissues, which is expected to further improve the applicability and long-term effectiveness of conservation strategies for *C. songaricum* [5]. Beyond species-specific insights, this work establishes a generalizable framework for studying metabolic specialization and environmental adaptation in other desert-adapted parasitic or succulent plants. By revealing how tissue-specific metabolism translates environmental signals into adaptive phenotypes, this study opens new avenues for integrative research linking metabolomics, ecophysiology, and conservation biology.

## Supporting information

S1 Table Sample collection information. This file is in XLSX format and contains detailed information of 33 sampling sites in this study, including sample number, sampling site, longitude, latitude, altitude (m) and remarks.
(XLSX)

S2 Table Reference and basis for soil sample determination methods. This file is in XLSX format and contains the reference and basis for the determination methods of 28 soil sample detection indicators, including detection indicator items and their corresponding standard method references.
(XLSX)

S3 Table Reference and basis for water sample determination methods. This file is in XLSX format and contains the reference and basis for the determination methods of 17 water sample detection indicators, including detection indicator items and their corresponding standard method references.
(XLSX)

S4 Table Amino acid content in *C. songaricum* stems. This file is in XLSX format and contains the detection data of 17 amino acid contents in 33 *C. songaricum* stem samples, including sample number and the content of each amino acid.
(XLSX)

S5 Table Amino acid content in *C. songaricum* epidermis. This file is in XLSX format and contains the detection data of 17 amino acid contents in 33 *C. songaricum* epidermis samples, including sample number and the content of each amino acid.
(XLSX)

S6 Table Soil physicochemical property test results. This file is in XLSX format and contains the test data of 27 physicochemical indicators for 33 soil samples, including sample number, heavy metal content, pH value, electrical conductivity, macroelement content, organic matter, and available nutrient content.
(XLSX)

S7 Table Water sample physicochemical property test results. This file is in XLSX format and contains the test data of 16 physicochemical indicators for 33 water samples, including sample number, pH value, electrical conductivity, ion content, heavy metal content, total salt content, and other water quality indicators.
(XLSX)
